# Comparison of Heavy Metal Pollution, Health Risk, and Sources Between Surface and Deep Layers for an Agricultural Region Within the Pearl River Delta: Implications for Soil Environmental Research

**DOI:** 10.3390/toxics13070548

**Published:** 2025-06-29

**Authors:** Zhenwei Bi, Yu Guo, Zhao Wang, Zhaoyu Zhu, Mingkun Li, Tingping Ouyang

**Affiliations:** 1 School of Geography, South China Normal University, 55 Zhongshan Road West, Guangzhou 510631, Chinalimk@m.scnu.edu.cn (M.L.); 2 Geological Survey of Guangzhou, Guangzhou 510440, China; gy03103301@163.com (Y.G.); wangzhao9333@163.com (Z.W.); 3 Guangzhou Institute of Geochemistry, Chinese Academy of Sciences, Guangzhou 510640, China; zhuzy@gig.ac.cn

**Keywords:** heavy metal pollution, health risk assessment, source analysis, surface soil, deep soil, agricultural area, the Pearl River estuary

## Abstract

During the past decades, agricultural soil heavy metal pollution has been becoming increasingly severe due to urbanization and industrialization. However, the impact of externally input heavy metals on deep soils remains unclear because most previous relevant research only focused on surface soils. In the present study, Concentrations of eight heavy metals (Cu, Zn, Ni, Pb, Cr, Cd, As, and Hg) were determined for 72 pairs of surface and deep soil samples collected from an agricultural region close to the Pearl River estuary. Subsequently, heavy metal pollution and potential health risks were assessed using the Geo-accumulation Index and Potential Ecological Risk Index, a dose response model and Monte Carlo simulation, respectively. Principal component analysis (PCA) and the positive matrix factorization (PMF) receptor model were combined to analyze heavy metal sources. The results indicated that average concentrations of all heavy metals exceeded their corresponding background values. Cd was identified as the main pollutant due to its extremely high values of *I_geo_* and *Er*. Unacceptable potential heavy metal non-carcinogenic and carcinogenic risks indicated by respectively calculated HI and TCR, higher than thresholds 1.0 and 1.0 × 10^−4^, mainly arose from heavy metals As, Cd, Cr, and Ni through food ingestion and dermal absorption. Anthropogenic sources respectively contributed 19.7% and 38.9% for soil As and accounted for the main contributions to Cd, Cu, and Hg (Surface: 90.2%, 65.4%, 67.3%; Deep: 53.8%, 54.6%, 56.2%) within surface and deep layers. These results indicate that soil heavy metal contents with deep layers were also significantly influenced by anthropogenic input. Therefore, we suggest that both surface and deep soils should be investigated simultaneously to gain relatively accurate results for soil heavy metal pollution and source apportionments.

## 1. Introduction

As the primary medium for energy flow and material circulation in nature [1], soil plays a pivotal role in sustaining ecosystems and supporting human development [2]. However, under escalating anthropogenic pressures from urbanization, industrialization, and agricultural activities, heavy metals are increasingly permeating soil ecosystems through pathways such as atmospheric deposition, surface runoff, and subsurface migration [3]. Rapid economic growth, population surges, and intensified production levels have contributed to a marked escalation of soil pollution [4]. Heavy metals, due to their environmental persistence, bioaccumulation, and toxicity [5], pose significant ecological risks. These contaminants have complex anthropogenic sources and are not eliminated within short timescales [6]. Furthermore, this issue is further compounded by the enrichment of heavy metals in agricultural soils, posing imminent threats to soil ecology, food safety, and human health because of heavy metal enrichment within the food chain [7]. Therefore, comprehensive studies encompassing source identification, contamination assessment, and health risk evaluation are critical prerequisites for implementing effective pollution monitoring and ensuring sustainable agricultural soil utilization.

Currently, the sources of heavy metals in agricultural soils are multifaceted, and the pollution characteristics of different regions are distinct. A pivotal area of research is the exploration of heavy metal contamination and its sources in agricultural soils, which exhibit refinement characteristics [8]. In relevant studies, researchers have primarily investigated surface soils as the dominant zone of heavy metal contamination. Geochemical data from deeper soil layers have been investigated only in a limited number of studies [9], and overall, comparative analyses of heavy metal sources across different soil layers are lacking. The exclusive reliance on surface soil monitoring might lead to the erroneous conclusion that the area is less contaminated, thereby ignoring the significant risks lurking in deep soils. Additionally, previous studies [10] have demonstrated a significant positive correlation between the concentration of heavy metals in agricultural soils and their presence in crops. Given that soil is the basis for food production and that the sources of heavy metals in different soil layers remain unclear, and that assessment results may be uncertain or biased, it is essential to account for the ecological and potential human health risks caused by heavy metal pollution in agricultural surface and deep soils [11].

In the Pearl River Delta (PRD), decades of rapid urbanization have led to numerous industrial and mining operations within urban and agricultural areas [2]. The upstream discharge of pollutants and the excessive application of fertilizers and pesticides significantly exacerbate ecological risks and pose threats to human health [12,13,14]. Research indicates that the mean concentrations of several heavy metals in agricultural soils within the PRD exceed local baseline levels, with notably elevated levels of As and Pb compared with those in other regions [15]. Cd has emerged as the most prevalent contaminant in both soils and crops, representing the heavy metal with the highest potential ecological risk among common pollutants [16]. Investigations into the potential health risks associated with agricultural soils reveal that the food ingestion is one of the primary pathways through which soil heavy metal pollution poses a threat to human health and that rice and maize pose the greatest health risks from heavy metals in the PRD [17]. The Geo-accumulation index (*I_geo_*) and potential ecological risk index (*RI*) have been widely used in evaluating soil heavy metal pollution and assessing ecological risk [18,19], and each assessment method has unique characteristics and limitations. Thus, for comprehensive analyses, it is necessary to integrate multiple evaluation methods for assessing regional pollution levels and ecological risks [20]. A general health risk assessment includes the exposure routes of hand-to-oral ingestion, dermal absorption, and inhalation, which are the three common pathways by which soil heavy metals enter the human body [21]. In studies on agricultural soils, special emphasis should be placed on the link between daily food ingestion and heavy metal accumulation [22]. Furthermore, deterministic evaluation methods are prone to deviations due to individual differences; therefore, probabilistic risk assessment methods such as Monte Carlo simulations must be adopted to mitigate uncertainties in the results [23].

Identifying heavy metal sources is the primary focus in addressing heavy metal pollution in agricultural soils [21]. Common methods for soil heavy metal source apportionment include multivariate statistical analysis [24], receptor modes [25], and geostatistical analysis [26]. Receptor modeling has emerged as the primary approach for quantitatively analyzing soil heavy metal pollution [27]. Positive matrix factorization (PMF) was originally employed for the analysis of atmospheric pollution sources [28], which offers advantages such as nonnegative constraints and facilitates the correlation of heavy metal concentrations with potential sources in the surrounding environment through spatial analysis, which has been extensively utilized in agricultural soils [29]. Currently, PMF is frequently employed in published studies to differentiate between natural and anthropogenic sources and to assign sources to surface layers according to the layer characteristics [30]. Notably, heavy metals are mobile and susceptible to migration to deeper layers under the influence of long-term anthropogenic activities [31]. Irrigation and other human activities have resulted in more active heavy metal accumulation and transport in agricultural soils in the 0–2 m depth range [32,33]. Conventional source analysis does not account for some downward-migrating heavy metals, and heavy metals are believed to be derived from natural sources when their concentrations are comparable to the regional background levels [29], even if the elements are input from anthropogenic sources. In such cases, it is probable that the results of the surface source analysis will not accurately reflect the actual sources of heavy metals in the region. Nevertheless, previous studies have focused predominantly on surface-level soil source analysis, prioritizing regional pollution on a horizontal scale [12]. Few studies have discussed the impact of the downwards transport of heavy metals on source apportionment as well as related issues concerning the underestimation of source contributions and the misattribution of heavy metal sources.

Accordingly, in this study, we selected an estuarine agricultural zone in the Pearl River Delta as the study area. Samples of surface soil (0–20 cm) and deep soil (150–200 cm) were collected, and pollution evaluation and risk assessment were conducted. The main objectives were (1) to explore the differences in heavy metal migration and concentration in surface and deep soil layers; (2) to evaluate the potential risks to human health associated with heavy metals at varying soil depths; and (3) to contrast the sources of heavy metals in surface and deep soils to improve the regional identification of heavy metal sources. This study enhances the precision of source identification and offers invaluable insights into regional heavy metal traceability, which is important for improving the quality of agricultural soils.

## 2. Materials and Methods

### 2.1. Study Area

**Figure 1:**
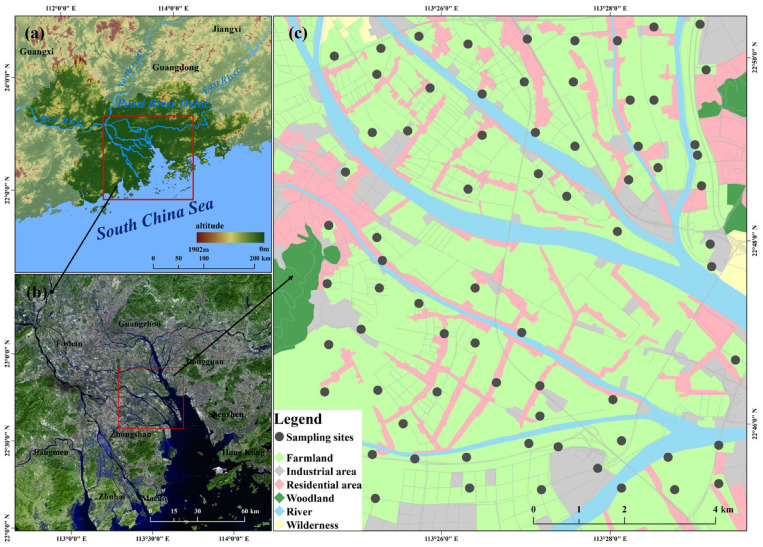
Location of the study area and distribution of sampling sites. (**a**) Water system and elevation of the PRD; (**b**) the spatial Location and water system of the study area within the PRD; (**c**) spatial distribution of sampling sites and land use of the study area.

The Pearl River Delta (PRD), where the West River, North River, and East River converge in China, boasts abundant water resources and diverse material sources (Figure 1a). The PRD, with an intricate river network and abundant water resources, has primarily undergone agricultural development. Since the implementation of the reform and opening-up policy in China, numerous industrial facilities, including those for electroplating, metallurgy, and building materials, have developed across agricultural regions within the PRD (Figure 1c) due to rapid economic advances, industrial development, and population growth over the last several decades [14]. Given the unique geographical position and complexity of human activities, the PRD epitomizes a typical agricultural zone influenced by urbanization and industrialization. To compare heavy metal pollution between surface and deep soils within river network agricultural areas, the Lingshan Agricultural Area (22°44′0″ N~22°52′0″ N, 113°24′0″ E~113°30′0″ E) (Figure 1c), characterized by flat terrain and located south of the PRD, was selected for sample collection. Owing to pedogenesis under warm and humid subtropical climates and a long history of rice cultivation, paddy soil and lateritic red soil, which developed from loose Quaternary accumulations, predominate the surface and deep soils within the study area, respectively.

### 2.2. Sample Collection and Heavy Metal Content Measurements

In this study, 72 sampling sites were selected from agricultural land. To ensure the representativeness of the samples, the distribution of the sampling sites encompassed the entire study area. A diverse range of crop types, such as fruit and leafy, root, and inflorescence vegetables, are included. Soil samples were collected from the same sampling site at two depths: 0–20 cm (Surface soil) and 150–200 cm (Deep soil). A total of 144 samples were ultimately obtained. The collection of deep samples reached the parent material layer of the study area. The collection process was based on specification of regional eco-geochemistry assessment. The 0–20 cm soil was excavated by shovels from the center and the four vertices surrounding the sampling point. The soil was then mixed and used as the surface soil sample for that sampling point. The initial stage involved soil drilling to a depth of 150 cm via a compact drill rig. This was followed by the replacement of the alloy bit and the attachment of a thin-wall soil sampler to the aforementioned rig through the spiral drill pipe to allow for the collection of deep soil samples between 150 and 200 cm. All samples were placed in plastic Ziplock bags in the field. Upon completion of sample collection in the field, the samples were transported to the laboratory for air-drying at room temperature, and any visible gravel or plant roots were removed [34].

Heavy metal concentration measurements were performed at the ALS Minerals-ALS Chemex Laboratory, Guangzhou, China. Chemical analyses were performed in strict accordance with the DZ/T0279-2016 Regional Geochemical Sample Analysis Standard for the eight heavy metals [35]. The material was sieved through a 74 μm mesh and ground into a powder for elemental measurements. Using the aqua regia water bath digestion method, aqua regia was prepared by mixing 3 volumes of hydrochloric acid with 1 volume of nitric acid, then the mixture was diluted with an equal volume of water. A total of 0.2 g of ground soil sample was transferred into a colorimetric tube, 10 mL of aqua regia was added, and digestion was performed in a boiling water bath for 2 h. The mixture was shaken every 30 min during digestion. After cooling, the solution was diluted to the mark and mixed thoroughly. After acid decomposition by the aqua regia, heavy metal concentrations were determined using an inductively coupled plasma mass spectrometry (ICP-MS, Agilent 7700x (Agilent Technologies., Santa Clara, CA, USA); the detection limits for Cu, Zn, Ni, Pb, Cr, Cd, As, and Hg were 0.01, 0.1, 0.04, 0.005, 0.01, 0.001, 0.01, and 0.004 μg/g, respectively). The accuracy of the analytical procedure was monitored during the process using the GSS series of national standard substances. Blank samples, duplicate samples and standard substances were inserted into each test batch for quality control, which was carried out in strict accordance with the Environmental Quality Standard for Soils [36]. The spiked recoveries of each heavy metal were controlled to be in the range of 90–110%.

### 2.3. Data Analyses

#### 2.3.1. Heavy Metal Pollution Assessment

Although many pollution indices are widely used for soil heavy metal pollution assessment, the focus and adaptation methods for each index are quite different [30]. To better evaluate heavy metal accumulation in soils compared with their geochemical background and compare the differences between surface and deep soil heavy metal pollution, the Geo-accumulation index (*I_geo_*) was used for soil heavy metal pollution assessment [37]. In addition, considering the toxicity of heavy metals, the potential ecological risk index (*RI*) was used to calculate the ecological risk caused by soil heavy metal pollution [38]. The definitions, calculation formulas and classification criteria of these indices are introduced in detail and listed in the text of the Appendix A.1 and Table A2.

#### 2.3.2. Health Risk Assessment

The widely used dose response model for potential human health risk assessment proposed and revised by the USEPA [39] was employed in the present study to quantify the potential health risks caused by soil heavy metals [40]. Overall, three main exposure pathways, hand-to-oral ingestion, dermal absorption, and inhalation, should be considered for soils [39]. For agricultural soils, heavy metal ingestion through the consumption of agricultural products must be considered [41]. Therefore, crop heavy metal concentrations were determined for each area of farmland at the respective sampling sites by employing the bioconcentration factors of crops cultivated in the region to assess the potential health risk of the food to humans (Table A3). Currently, it is difficult to accurately quantify the effects of heavy metal pollution in deep soils on human health through direct exposure pathways. Nevertheless, the results of the mentioned model can provide an approximate reference that highlights the potential risks present in deep soils. The calculation formulas and corresponding parameter settings for potential human health risk assessment are shown in the text of the Appendix A.3 and in Table A3, Table A4, and Table A5, respectively.

Considerable uncertainty and variability in the results of health risk assessments can arise due to the complexity of pollution sources and the physical conditions and heavy metal intake rates of different populations, coupled with the use of fixed parameters and deterministic methods [42]. To reduce uncertainties, a Monte Carlo simulation was employed via the Oracle Crystal Ball tool [43]. Under the framework of the probabilistic simulation, specific variables in the health risk assessment are replaced with probability distribution functions with normal or triangular distributions. In addition, 10,000 iterations were performed to calculate the carcinogenic and noncarcinogenic risks caused by each heavy metal via each exposure pathway.

#### 2.3.3. Source Analysis

Multivariate statistical analysis and receptor models are most frequently employed for source analysis. In multivariate statistics, principal component analysis (PCA) provides the weights of each component and identifies the components affecting the distribution of heavy metals by reducing the number of factors [44]. The Kaiser-Meyer-Olkin (KMO) measure was employed to retain factors with eigenvalues exceeding 1, followed by a varimax rotation method. This method was combined with a correlation matrix and cluster analysis for the source identification of heavy metals [3]. PCA can facilitate initial identification of the number of sources of heavy metals, distinguishing between natural and anthropogenic sources [45]. In the next step, the positive matrix factorization (PMF) method was employed to validate the PCA results and further quantify the contributions of different sources to the overall concentrations of heavy metals.

As a receptor model, the PMF model is most widely used to identify sources and quantify their contributions because of its convenience and efficiency and its ability to quantify sources under nonnegative constraints and data uncertainty [46]. Although the emission inventory method can achieve high accuracy, it requires a large amount of basic data, which is time-consuming and costly to obtain [47]. Geostatistical analysis and multivariate statistical analysis can be used to identify sources only roughly and lack precise quantification of contributions [48]. Isotope ratio analysis requires stable isotopes and significant differences in isotopic composition between soil and sources. Therefore, this method is difficult to apply for complex contaminants [49].

Furthermore, the PMF is also a multivariate analysis tool for decomposing a sample-specific data matrix into a factor score matrix, a factor loading matrix, and a residual matrix [50]. This model relies solely on measured data for various heavy metals at sampling points and their corresponding uncertainty data [51]. Therefore, the PMF model was used for soil heavy metal source apportionment in the present study. The process uses two-dimensional sample data matrices and uncertainty matrices to separately analyze the source of heavy metals in surface and deep samples. The basic principles and calculation formulas of the PMF model are based on Paatero and Tapper [52], and the specific formulas and calculation steps are described in the main body of the Appendix A.2.

#### 2.3.4. Statistical and Spatial Analyses

Descriptive analysis of the heavy metal concentration and pollution assessment data was performed via IBM SPSS Statistics 25. The descriptive statistical results were subsequently displayed through graphics drawn via the R-4.0.3 programming language and Origin Pro 2021 software. To compare the soil heavy metal concentrations between surface and deep layers and identify the layer containing a high concentration of each heavy metal, the spatial distribution of heavy metal concentrations within surface and deep soils was modelled in three-dimensional space via a groundwater modelling system (GMS). Three interpolation methods, namely regularized, inverse distance weighting (IDW), and kriging, were used to obtain the spatial distributions of the soil heavy metal pollution index and ecological risk via ArcGIS 10.5 software.

## 3. Results and Discussion

### 3.1. Characteristics and Spatial Distribution of Heavy Metal Concentration

**Figure 2:**
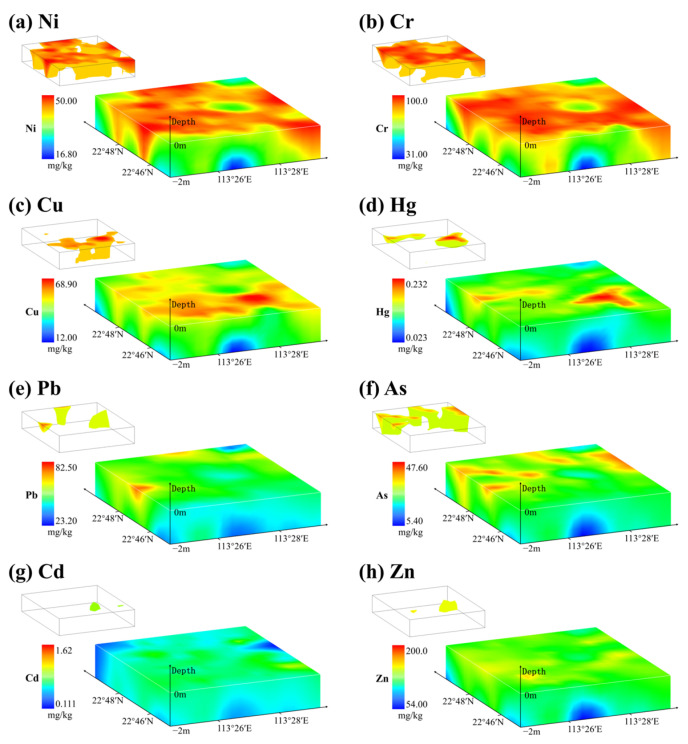
Spatial distribution of heavy metal concentrations in a three-dimensional space and distribution of areas with high concentration (top 25% of the concentration range).

**Figure A1:**
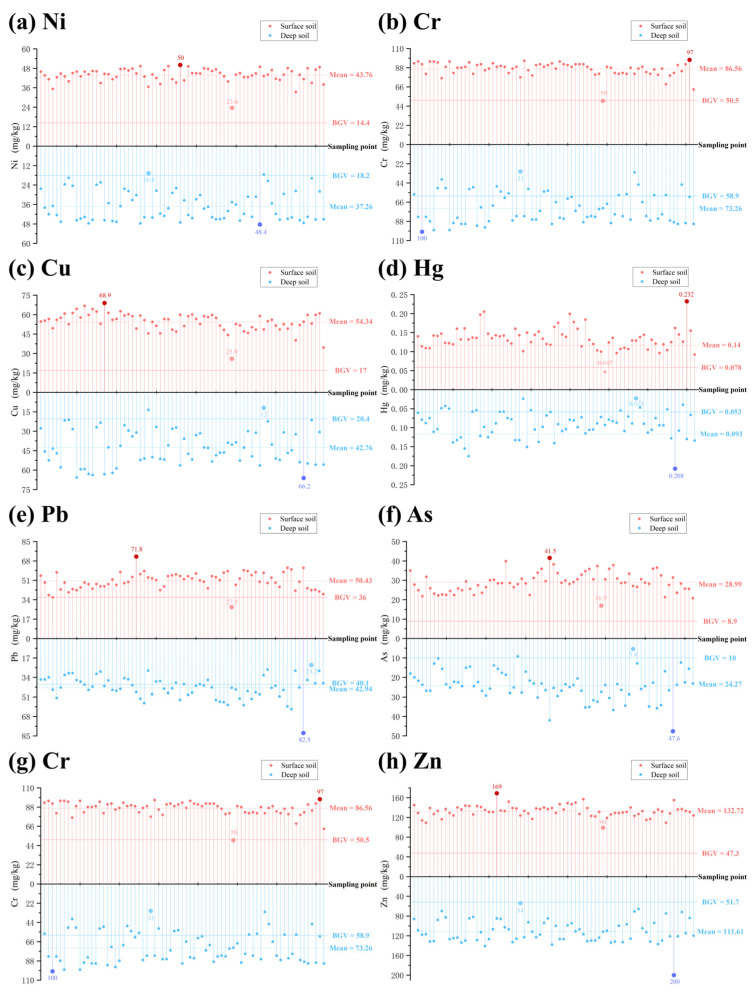
Measured heavy metal concentrations in deep and surface soils. (**a**) Ni, (**b**) Cr, (**c**) Cu, (**d**) Hg, (**e**) Pb, (**f**) As, (**g**) Cr, and (**h**) Zn. The solid blue and dashed pink lines present the background values of soil heavy metal contents for surface and deep layers in Guangdong province.

A summary of descriptive statistical results for heavy metal concentrations within soil samples and the background values of soil heavy metal contents for Guangdong province are listed in Table 1. In addition, spatial distribution and comparisons of soil heavy metal concentrations between surface and deep layers are shown in Figure 2. A detailed comparison of heavy metal concentrations between surface and deep soils for each sampling site is depicted in Figure A1.

The results listed in Table 1 indicate that the average concentrations of all measured heavy metals were higher than their corresponding background values for both surface and deep soils. Among the eight measured heavy metals, Cd exhibited the most significant deviation from its background value for soils (Figure A1). The average concentrations of Cd within surface and deep soils were 8 and 9 times higher, respectively, than their corresponding background values (Table 1). The average Cu, Zn, Ni, and As concentrations within surface and deep soils were more than three and two times greater than their corresponding background values (Table 1 and Figure A1). Fortunately, the average Pb, Cr, and Hg concentrations within surface and deep soils only slightly exceeded their background values (Table 1). Although the average and minimum concentrations of all measured heavy metals within deep soils were lower than those within surface soils, the maximum Zn, Pb, Cr, Cd, and As concentrations within deep soils were higher than those within surface soils (Table 1). Moreover, the spatial heterogeneity of heavy metal concentrations reflected by the CV tended to increase from the surface layer to deep soil layers (Table 1), indicating that soil heavy metal contents of both surface and deep layers within the study area are significantly affected by external factors.

According to the results illustrated in Figure 2, the spatial distribution trends of various heavy metals exhibited significant disparities. Notably, the Ni and Cr concentrations exhibited analogous spatial distributions, with high concentrations primarily appearing on the surface and covering a wide area. The research found that the road network covers nearly the entire area and has a spatial distribution similar to that of the Ni and Cr concentrations (Figure 1c). This result indicates a robust correlation between traffic and vehicle emissions and the aforementioned elements. Furthermore, high Cu and Hg concentrations existed in both surface and deep soils in the southern part of the study area (Figure 2c,d), with a distribution similar to that of the electromechanical and construction material factories in the region [2,14]. In contrast, high Pb and As concentrations in both surface and deep soils were clearly distributed in the northern regions of the study area (Figure 2e,f). No obvious difference in their contents was detected within surface and deep soils, suggesting a more homogeneous source of soil Pb and As [54]. The Cd and Zn concentrations within surface soils are generally higher than those within deep soils (Figure 2g,h). However, anomalously high values of Cd and Zn concentrations in deep soil were observed in the northeastern regions of the study area, which is situated adjacent to the Jiaomen watercourse and the primary creek of the village, with a large agricultural base [55]. The mentioned results may suggest that a close relationship between agricultural cultivation and irrigation activities and soil heavy metal accumulation in deep layers [56]. Furthermore, it can be deduced that the spatial distribution and heterogeneity of various heavy metals may be influenced by the mentioned anthropogenic activities.

### 3.2. Heavy Metal Pollution and Its Spatial Distribution

**Figure 3:**
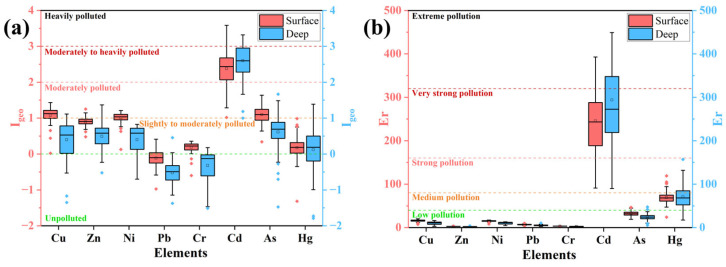
Statistical results of (**a**) Geo-accumulation index (*I_geo_*); (**b**) potential ecological risk index (*Er*) for each heavy metal for both surface and deep soils.

**Figure 4:**
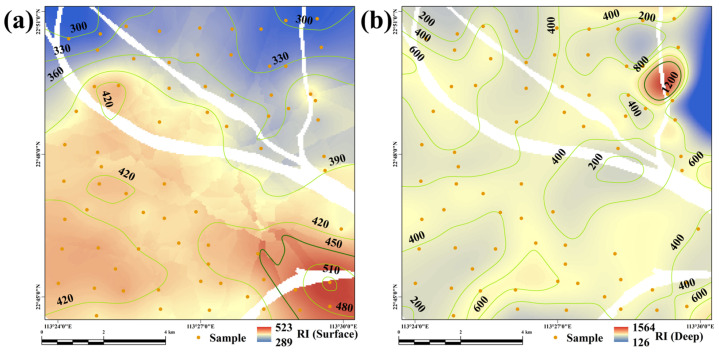
Spatial distribution of potential ecological risk index (RI) for (**a**) surface and (**b**) deep soils.

The calculated results of the Geo-accumulation index (*I_geo_*) and potential ecological risk index (*Er*) for each heavy metal are illustrated in Figure 3 for both surface and deep soils. Moreover, the spatial distribution of the calculated total potential ecological index (*RI*) is shown in Figure 4.

Research on agricultural surface soils by Gan et al. [57] and Enuneku et al. [58] revealed no obvious heavy metal contamination in the Yellow River Delta or Nigerian Delta. The heavy metals Cd, Cu, and Pb have been identified as major agricultural surface soil pollutants in the Yangtze River Delta and the North Nile Delta [59,60]. Moreover, Nguyen et al. [61] reported that anthropogenic As was the predominant pollutant in agricultural soils in the Red River Delta. However, compared to soil heavy metal pollution of agricultural regions within other deltas, heavy metal pollution of the study area exhibited different characteristics. For example, the *I_geo_* results indicated slight to moderate pollution levels of heavy metals Cu, Zn, Ni, As, and Hg within both surface and deep soils and Cr within surface soils (Figure 3a). Furthermore, previous research indicated that Cd and Pb are generally enriched in both cultivated and natural soils in many areas of the PRD [62], but only Cd showed a state of moderate to heavy pollution among the measured eight heavy metals due to its *I_geo_* results between two and three within both surface and deep soils in the study area (Figure 3a). No pollution was observed from heavy metal Pb within both surface and deep soils due to their *I_geo_* being lower than 0 (Figure 3a), which is consistent with the results reported by Xia et al. [13]. As shown in Figure 3b, low levels of Cu, Zn, Ni, Pb, Cr, and As pollution, indicated by their calculated Er lower than 40, appeared in both surface and deep soils. However, medium Hg and very strong Cd pollution were detected in both surface and deep soils (Figure 3b). Moreover, the calculated *I_geo_* and Er values of Hg and Cd for deep soils were greater than those for surface soils (Figure 3), indicating that more Hg and Cd had accumulated in deep soils.

It can be generally concluded from the calculated *RI* illustrated in Figure 4 that the spatial distribution of the *RI* was significantly different between surface and deep soils. Almost all surface soils within the study area presented potential strong ecological risk due to moderate heavy metal pollution, with an increasing trend from the northern to southern regions (Figure 4a). In the soil samples of the deep layer, the calculated *RI* values for most of them ranged from 300 to 600, indicating a strong ecological risk. Moreover, an abnormally extreme high-risk zone of deep soil with *RI* values higher than 1200 located in the northeastern part of the study area (Figure 4b) suggested a large amount of externally input heavy metals may be transported into deep soil layers. This zone was adjacent to a large agricultural base, where significant amounts of pesticides and fertilizers containing impurities have been utilized over an extended period. Moreover, the area was situated at the downstream end of the village creek and at a confluence point with the Jiaomen watercourse. The prevalence of heavy metal contamination and anthropogenic disturbances in both urban and rural ditches in the PRD resulted in soil heavy metal levels highly similar to those in neighboring ditches [54], and soil moisture also shows a notable positive correlation with heavy metals [63]. Upstream anthropogenic releases of heavy metals can easily flow downstream through rivers and ditches and eventually re-enter agricultural soils through irrigation [55]. The upstream passage traverses residential areas, waste stations, and intricate streets and highways, leading to the influx of significant amounts of heavy metals such as Cd, Cu, and Hg into the water body [64]. These heavy metals subsequently permeate deep layers into agricultural soils through irrigation, resulting in elevated concentrations of heavy metals in deeper soils. Furthermore, the deep soil samples have reached the parent material layer and the heavy metal contamination will affect the soil formation process, which will lead to the elevation of the regional soil heavy metal content.

Meanwhile, the 0–2 m layer in a soil profile constitutes an active zone for heavy metal transport and transformation [32]. In particular, for the agricultural region within South China, which is heavily influenced by anthropogenic activities, the 2 m range represents a soil layer of great human and ecological significance [33]. Variations in anthropogenic activities can cause differences in the input and transport rates of heavy metals, leading to their local accumulation in various zones [49]. As shown in Figure 3 and Figure 4, the comprehensive heavy metal pollution as well as the *I_geo_* and *Er* values of Cd and Hg were greater in deep soil than in surface soil, indicating that anthropogenic activities lead to the release of heavy metals into agricultural soils, which can then be transported downwards to deeper soil layers through long-term leaching due to irrigation [65]. Significant spatial disparities exist in agricultural soil heavy metal pollution due to different background values and agricultural activity intensities [66]. As a result, in previous soil contamination studies, researchers may have overlooked the process of heavy metal migration and accumulation between surface and deep layers, potentially leading to incorrect conclusions regarding overall regional pollution status and sources.

### 3.3. Potential Human Health Risks

We calculated the non-carcinogenic risk indices (HI) and the carcinogenic risk indi-ces (TCR) of various heavy metals across all exposure pathways. The descriptive statistical results of carcinogenic and non-carcinogenic health risks caused by heavy metals based on the Monte Carlo simulations are listed in Table A6 for surface and deep soils. Since the average values of HI and TCR respectively exceeded the thresholds of 1.0 and $1.0\times 10^{-4}$ for all populations, the residence within the study area may face noticeable non-carcinogenic hazards and carcinogenic risks caused by soil heavy metal pollution in both surface and deep layers. Furthermore, the HI and TCR levels for children are respectively much higher and lower than those for adults, suggesting that children within the study area may respectively face more and less significant potential non-carcinogenic hazards and carcinogenic risks caused by heavy metal pollution than adults.

**Figure 5:**
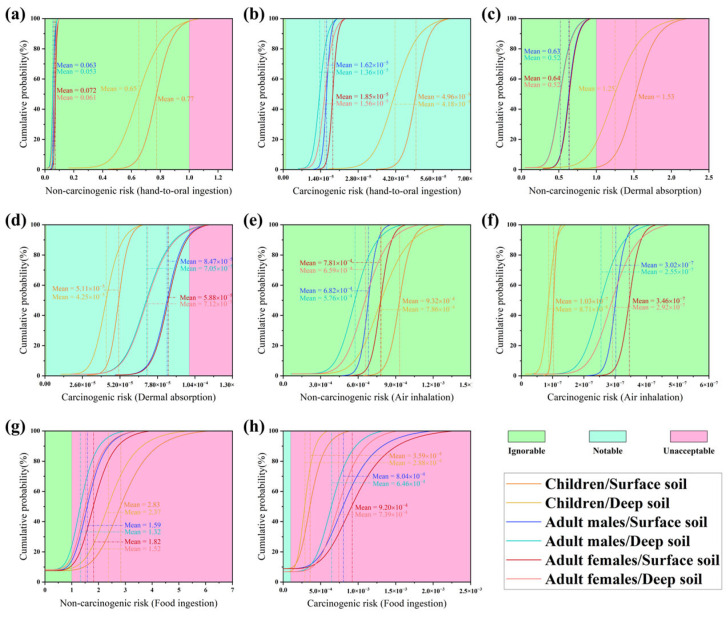
Cumulative probability curves of (**a**) HI and (**b**) TCR through hand-to-mouth ingestion; (**c**) HI and (**d**) TCR through dermal absorption; (**e**) HI and (**f**) TCR through air inhalation; (**g**) HI and (**h**) TCR through food ingestion, for different populations caused by soil heavy metals.

In addition, we obtained HI and TCR cumulative probability curves using calcula-tions from dose-response model and Monte Carlo simulations. As shown in Figure 5g,h, the HI and TCR values of 10% cumulative probabilities exposed through pathway food ingestion clearly exceeded the thresholds. The potential health risks exposed through pathway food ingestion were significantly greater than those through other exposure pathways, which differed from the findings of previous studies concerning potential human health risks caused by urban soil heavy metal pollution [67]. These results suggested more attention must be focused on the potential risks of heavy metal pollution in agricultural soils, because heavy metals could pose exceedingly high risks to humans once they are transferred from soil to crops [68]. The bioconcentration factors reflect the accumulation of heavy metals from the soil in the edible parts of plant tissues (leafy, fruit, root, and inflorescence vegetables, sugarcane, and bananas) and reveal the potential health risk of food to humans. Among the crops examined, inflorescence vegetables, sugarcane, and bananas demonstrated high transport efficiencies for heavy metals (Table A3). Although certain heavy metals are not vital nutrients for crops, they may accumulate in the crop’s edible tissues through ATP-dependent processes and transpiration [69]. Moreover, the study area serves as the primary sugarcane growing region in Guangzhou and a pivotal intensive sugarcane industrial base, accounting for 45.8% of the sampled agricultural land. The PRD boasts a well-developed sugar industry, which prevails as the primary avenue for sugarcane consumption, while direct consumption and pressing of raw juice for drinking are common methods of individual consumption. Additionally, leafy vegetables constitute 16.6% of important crops in the agricultural areas. Substantial industrial bases and a vast consumer market for cash crops such as bananas and inflorescence vegetables exist within the study area and in neighboring regions [70].

**Figure 6:**
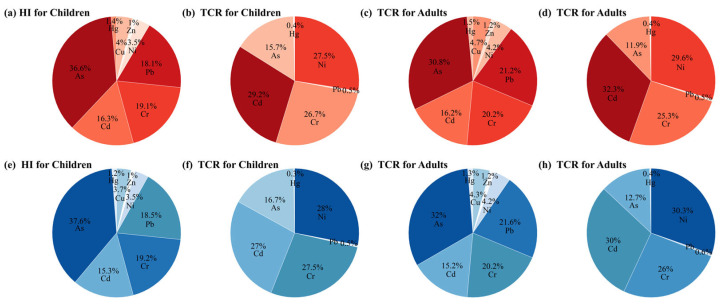
Contribution of each heavy metal to HI and TCR for different populations. (**a**–**d**) Surface and (**e**–**h**) deep soils.

The contribution of each heavy metal and every exposure pathway to HI and TCR for different populations are displayed in Figure 6. For both surface and deep soils, heavy metals As, Pb, Cr, and Cd were identified as the main contributors to non-carcinogenic risks. Meanwhile, heavy metals Cd, Ni, Cr, and As were the primary contributors to carcinogenic risks. As the primary contributor to non-carcinogenic risks, heavy metal As accounted for more than 30% of the non-carcinogenic risks for both children and adults because of its relatively high natural background concentrations and low reference doses [71]. Heavy metals Cd and Ni are classified as Group I carcinogens by international cancer research agencies [72]. Heavy metals Cd and Ni, with about 30% contribution for all populations (Figure 6), were identified as the primary contributors to total carcinogenic risks in both surface and deep soils. The high contribution of heavy metal Cd to TCR is due to its extremely high concentration (Table 1) as well as its high toxic toxicity coefficient. According to previous research, heavy metal Ni can be transformed to water-insoluble Ni compounds, such as Ni3S2 and NiO [72,73]. As a result, slope factors (SFs) of Ni through every exposure pathway are significantly higher than those of other heavy metals, resulting its high contribution to TCR.

### 3.4. Sources of Soil Heavy Metals

**Figure 7:**
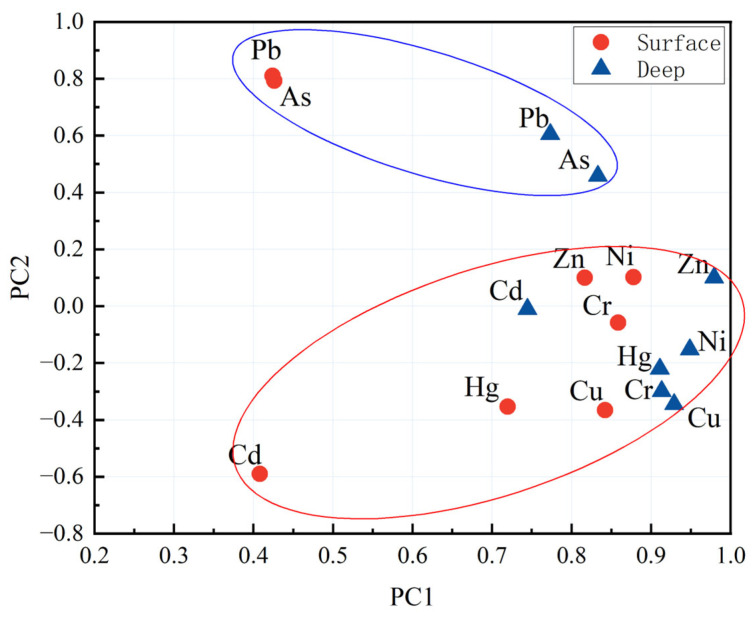
Distribution of component loadings of heavy metals in a rotated space for surface and deep soils.

The results of the correlation analysis indicated that the eight measured heavy metals were significantly correlated with each other (Table A7). Therefore, the main factors influencing all measured heavy metals could be identified from factor analysis via principal component analysis (PCA) [73]. Based on the Kaiser-Meyer-Olkin (KMO) and Bartlett sphericity tests (Surface: KMO = 0.72 > 0.5, *p* < 0.001; Deep: KMO = 0.82 > 0.5, *p* < 0.001), PCA retained two principal components with eigenvalues greater than one, PC1 accounted for 49.1% of total variance in the surface layer and 77.9% in the deep layer, while PC2 explained 23.9% and 10.8% of total variance in these layers, respectively. The distribution of component loadings of each heavy metal in a rotated space is illustrated in Figure 7 for both surface and deep soils.

Heavy metals Pb and As, which were significantly positively correlated with each other (Figure 7, Table A7), were significantly positively related with both PC1 and PC2, indicating that these two heavy metals within both surface and deep soils are influenced by the two identified factors. Moreover, the loadings of Pb and As were much greater on PC2 than other heavy metals within surface and deep soils (Figure 7), implying that PC2 was the main factor influencing these two heavy metals. Heavy metals Zn, Ni, Cr, Cu, and Hg within both surface and deep soils, which were significantly positively correlated with each other (Table A7), were positively related with PC1 and showed no obvious correlation with PC2 (Figure 7), indicating that these heavy metals within both surface and deep soils were impacted by a same factor. Heavy metal Cd was negatively and positively related with PC2 and PC1 in the surface and deep soils, respectively (Figure 7).

As weathering and erosion of rock minerals are the primary mechanisms for the release of heavy metals [74], parent materials and geochemical anomalies are considered as typical natural sources of soil heavy metals. In a natural state, the abundance of heavy metals in the soil system generally remains low, close to background values [75]. Anthropogenic sources, including industrial production, urban construction, traffic emissions, fertilizer and pesticide application, and ploughing and irrigation practices, have been shown to be notable origins of heavy metals within agricultural soils [59]. Some previous studies have indicated that deep soils are influenced primarily by geological background and geographical conditions in the PRD [76]. During long-term evolution of the PRD, loose Quaternary accumulations supplied the original heavy metals to deep soils. Due to the weathering of parent materials during the soil-forming processes, geological condition became the main contributor of heavy metals in surface soils [77]. In addition, a large number of arsenic-bearing minerals are transported to the Pearl River estuary by river systems. As a result, heavy metal As exhibited a pronounced high depositional background in the study area [55,78]. Moreover, the average concentration of Pb in both surface and deep soils was closely aligned with the natural background value within the region (Table 1). Thus, the PC1 and PC2 should be considered as anthropogenic and natural sources, respectively.

Although the results of PCA gave some evidence to extract the main factors that influenced soil heavy metals, the quantitative contributions of different factors remained unclear. The PMF model was used in the present study to quantify the contributions of natural and anthropogenic sources, which are regarded as the two main sources of soil heavy metals [79], to heavy metal contamination in surface and deep soils. A comparison between the factor loading matrix and the factor profile matrix reveals a notable similarity between the results of PCA and PMF (Table 2). In the surface layer, the contribution of Factor 2 to Pb and As is significantly higher than that to other heavy metals, which aligns with the performance of PC2. Meanwhile, both Factor 1 and PC1 show relatively high contributions to the other heavy metals. In the deep layer, Factor 2 and PC2 still exhibit substantial contributions to Pb and As. In addition, Factor 1 has decreased effect on three elements, Zn, Ni, and Cr.

**Figure 8:**
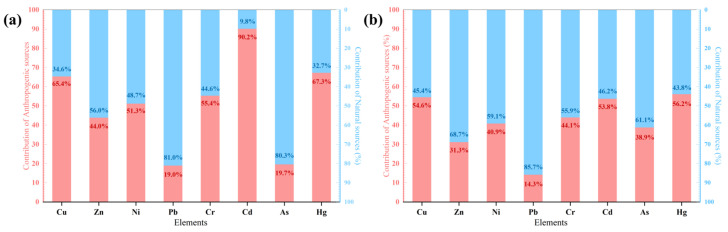
Contribution of natural and anthropogenic sources to each heavy metal. (**a**) Surface soil; (**b**) deep soil.

The PMF results in Figure 8 more clearly show the contribution of natural and anthropogenic sources to each heavy metal. From Figure 8, a factor contributed 80.3% and 81.0%, respectively, to the total sources of As and Pb in surface soils and contributed 61.1% and 85.7%, respectively, to the sources of these two elements in deep soils (Figure 8). Combined with the PCA results, it can be posited that the factor is natural sources. Another factor identified from the PMF model accounted for 65.4%, 51.3%, 55.4%, and 67.3% of the Cu, Ni, Cr, and Hg in surface soils, respectively (Figure 8). The PCA results revealed that Cu, Ni, Cr, and Hg shared common sources. In addition, the PMF results indicated that the factor significantly contributed to Cd, with 90.2% and 53.8% contributions to surface and deep soils, respectively (Figure 8). In addition, the concentrations of Cd within surface and deep soils significantly differed from those of natural Pb and As (Figure A1). The factor can therefore be identified as anthropogenic sources.

A comparison of PCA source analysis results between surface and deep soil samples from the same sampling sites revealed that one-third of the sampling sites showed opposite trends in the performance of the PC1 scoring factor, and 19% of the sites in PC2 showed opposite characteristics in the surface and deep layers. This suggests that there are some areas where the dominant sources of heavy metal content in the surface and deep layers are not consistent. Comparing the results of PMF between the surface and deep layers, the proportion of anthropogenic sources of As in deep soils was 38.9%, which was significantly greater than the 19.7% found in surface soils (Figure 8). This indicates that anthropogenic sources of heavy metals are transported, reducing their presence in the surface soil but increasing their concentrations in deep soil. This process reduces the expression of anthropogenic factors in surface soils, thereby obscuring the actual sources of heavy metals in surface soil studies [65]. In this case, an evaluation of pollution sources and heavy metal source apportionment in the surface soil alone would result in an underestimation of the contributions of heavy metals from anthropogenic sources and the contamination levels of the region as a whole. Furthermore, this would result in the incorrect tracing of heavy metals.

**Figure A2:**
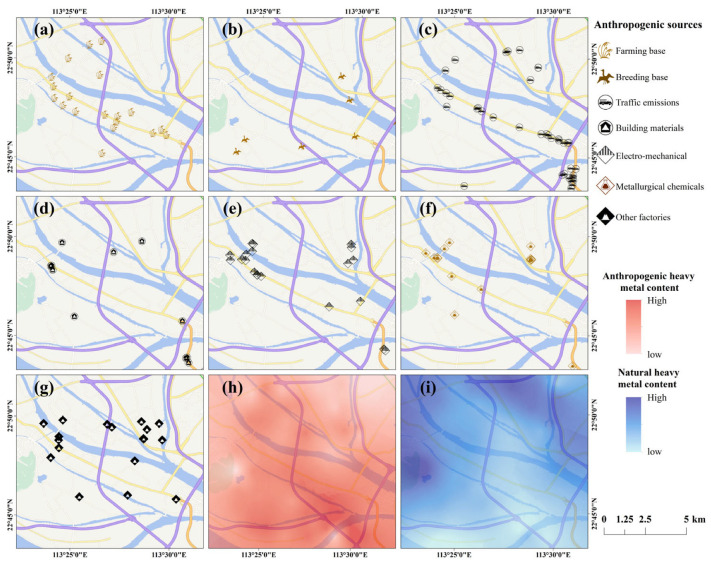
Spatial location of anthropogenic sources as well as anthropogenic and natural heavy metal contents in the study area. (**a**) Farming base, (**b**) breeding base, (**c**) traffic emissions, (**d**) building materials, (**e**) electro-mechanical, (**f**) metallurgical chemicals, (**g**) other factories, (**h**) anthropogenic heavy metals, (**i**) natural heavy metals.

Previous studies on the PRD suggested that the concentration of heavy metals Cu, Ni, Cr, and Hg within surface soils may be influenced mainly by anthropogenic activities [55,76]. In the study area, numerous industrial facilities, including those for electroplating, metallurgy, and building materials, are distributed near agricultural areas (Figure A2). Elevated concentrations of Cu have been reported in dust emitted from the disassembly or combustion of electronic products in electromechanical factories [19]. Hg is emitted during the combustion of fossil fuels, wastewater treatment, and the production of building materials in brick manufacturing facilities [18]. In addition, the road network covering the area is a substantial source of solid particulate matter containing Cr, Ni, and Zn [12]. Moreover, the study area is situated within an agricultural production zone, where the use of fertilizers and pesticides is much greater than the national average, and a considerable amount of Cd is present in phosphate fertilizers in the form of impurities [2,46]. Consequently, anthropogenic activities in the fields of industrial production, transportation, and agriculture have had a considerable impact on both surface and deep soils.

Comparing the results of anthropogenic sources of PMF, the contribution of anthropogenic sources to deep soils exceeded 40%, a result comparable to their 51.5% contribution in surface soils. In particular, three elements, Cd, Cu, and Hg, even presented contributions of over 50% from anthropogenic sources in surface and deep soils (Figure 8). Notably, Cd is readily soluble in water and has high bioavailability and mobility [31]. In addition, the study area has abundant water resources and intersecting ditches, which facilitates efficient drainage and irrigation operations and the downward migration of heavy metals [54]. Moreover, the study found that intensive agricultural practices involve the use of Hg-containing sewage water for irrigation, Cd-containing phosphorus fertilizer, and Cu-containing livestock manure for cultivation. Furthermore, industrial activities result in the release of substantial amounts of Cu- and Hg-containing waste particles, which subsequently enter the soil through atmospheric deposition [18,32,76]. Leaching and preferential flow are important mechanisms for the downward transport of Cd, Cu, and Hg after they enter the soil [33] and are important drivers of accelerated downward transport [12]. Active anthropogenic activities and frequent water movement may result in the significant transport of elements that would otherwise accumulate in the surface layer to the lower layers.

Generally, externally input heavy metals tended to be accumulated primarily in the surface soil layer [63]. Deep soil is often utilized as the background and foundation for quantifying the diffusion of heavy metals from surface soil [76]. Nevertheless, due to high levels of anthropogenic activities, external inputs of heavy metals result in nonnegligible contributions to elemental contents in deep soils. The results of the source analysis shown in Figure 8b also confirm that deep soils were significantly influenced by anthropogenic activities.

## 4. Conclusions

In this study, heavy metal concentration, pollution, and risk assessments were carried out for both surface and deep soils within an agricultural region in the PRD. The following conclusions were drawn.

Concentrations of all eight measured heavy metals in both surface and deep soils exceeded their corresponding background values for Guangdong province. The ecological risk index results indicated strong heavy metal pollution in surface soils and strong to very strong and even extreme heavy metal pollution in deep soils. The high heavy metal pollution levels appeared in the southeast and northeast regions of the study area for surface and deep soils, respectively. In addition, the main pollutants in both surface and deep soils were Cd and Hg. The residents within the study area may suffer significant potential health risks as caused by soil As, Cd, Cr, and Ni pollution, mainly through the exposure pathway of food ingestion.

The source apportionment results indicated heavy metals As, Pb, and Zn within both surface and deep soils originated from natural sources. However, anthropogenic sources such as agricultural, industrial, and traffic sources accounted for about 90.2% of Cd, 67.3% of Hg, 65.4% of Cu, 55.4% of Cr, and 51.3% of Ni in surface soils. In addition, the contribution of these anthropogenic sources to heavy metals Hg, Cd, and Cu in deep soils were higher than the contribution of natural sources. Notably, the contribution of anthropogenic sources to heavy metal As in deep soils was much higher than that in surface soils.

The above results imply that there is also a contribution from anthropogenic sources to the heavy metal content of deeper soils and that this contribution from anthropogenic sources cannot be detected by surface soil monitoring. Therefore, our study suggested that simultaneous monitoring and comparison of surface and deep soil heavy metals can provide new research perspectives for heavy metal source analysis.

## Figures and Tables

**Table 1:** A summary of descriptive statistical results for heavy metal concentrations (mg/kg) within surface (0–20 cm) and deep (150–200 cm) soils.

Heavy Metals	Range		Mean		CV		BGV	
	Surface	Deep	Surface	Deep	Surface	Deep	Surface	Deep
Cu	25.90–68.90	12.00–66.20	54.34	42.76	0.13	0.31	17	20.4
Zn	99.00–169.00	54.00–200.00	132.72	111.61	0.09	0.21	47.3	51.7
Ni	23.60–50.00	16.80–48.40	43.76	37.26	0.10	0.24	14.4	18.2
Pb	27.50–71.80	23.20–82.50	50.43	42.94	0.14	0.23	36	40.1
Cr	50.00–97.00	31.00–100.00	86.56	73.26	0.09	0.24	50.5	58.9
Cd	0.17–1.01	0.11–1.62	0.46	0.36	0.32	0.49	0.056	0.037
As	16.90–41.50	5.40–47.60	28.99	24.27	0.17	0.31	8.9	10
Hg	0.05–0.23	0.02–0.21	0.14	0.09	0.21	0.37	0.078	0.053

Note: CV represents Coefficient of variation; BGV represents Background values of Guangdong province from China National Environmental Monitoring Centre [53].

**Table 2:** PCA factor loading matrix and the PMF factor profile matrix (conc. of species).

Heavy Metals	Surface		Deep		Surface		Deep	
	PC1	PC2	PC1	PC2	Factor 1	Factor 2	Factor 1	Factor 2
Cu	0.842	−0.366	0.929	−0.345	35.60	18.80	23.128	19.216
Zn	0.817	0.099	0.979	0.1	58.20	74.20	34.885	76.701
Ni	0.878	0.102	0.949	−0.153	22.50	21.40	15.193	21.952
Pb	0.424	0.81	0.773	0.605	9.62	40.90	6.119	36.726
Cr	0.858	−0.058	0.913	−0.299	47.90	38.60	32.276	40.958
Cd	0.408	−0.589	0.744	−0.011	0.41	0.04	0.183	0.158
As	0.426	0.793	0.833	0.458	5.64	23.10	8.796	13.827
Hg	0.72	−0.353	0.911	−0.221	0.09	0.04	0.05	0.039

## Data Availability

The original contributions presented in this study are included in the article. Further inquiries can be directed to the corresponding author.

## Abbreviations

The following abbreviations are used in this manuscript:

*I_geo_*	Geo-accumulation Index
*Er*&*RI*	Potential Ecological Risk Index
PCA	Principal component analysis
PMF	Positive matrix factorization
PRD	Pearl River Delta
HI	non-carcinogenic risk indices of various heavy metals across all exposure pathways
TRC	carcinogenic risk indices of various heavy metals across all exposure pathways

## Appendix A

### Appendix A.1. Geo-Accumulation Index

The Geo-accumulation Index (*I_geo_*) is founded on the variability of heavy metal distribution characteristics resulting from natural diagenesis and geological processes [80]. It quantifies the disparity in soil heavy metal concentration from the original background value under anthropogenic influences [81], thus serving as a visual indicator for assessment of the heavy metal enrichment degree. The following formula is widely used for the calculation of *I_geo_* for each heavy metal.

(A1) $$I_{geo}=\log_{2}\left(\frac{C_{i}}{kB_{i}}\right)$$

where, $C_{i}$ and $B_{i}$ denote the measured concentration and background value of heavy metal *i*. The soil background values are derived from the heavy metal background values of different soil horizons in Guangdong Province, as published by the China National Environmental Monitoring Centre [53]. The background concentrations of heavy metals in the surface and deeper layers are shown in Table A1. Additionally, k (1.5) represents a dimensionless rock variance coefficient [82], employed to adjust for regional discrepancies in background values. The evaluation criteria for assessing pollution levels are listed in Table A2.

### Appendix A.2. Potential Ecological Risk Index

The Potential Ecological Risk Index method proposed by Håkanson [83] is primarily concerned with the toxicological assessment of heavy metals, taking into account the synergistic effects of different heavy metals on biotoxicity and multiple hazardous elements. The Potential Ecological Risk Index of each heavy metal as well as the total values of a sample can be calculated using the following formula.

(A2) $$RI=\sum_{i=1}^{n}E_{r}^{i}=\sum_{i=1}^{n}T_{r}^{i}\times P_{i}=\sum_{i=1}^{n}T_{r}^{i}\times \frac{C_{i}}{B_{i}}$$

where $E_{r}^{i}$ denotes the potential ecological hazard posed by individual metal *i*, while $T_{r}^{i}$ represents the corresponding toxicity coefficient (Cu = Ni = Pb = 5, Zn = 1, Cr = 2, Cd = 30, As = 10, Hg = 40). $C_{i}$ and $B_{i}$ respectively signify the measured concentration and corresponding background values of heavy metal *i*. The evaluation criteria for assessing pollution levels are detailed in Table A2.

### Appendix A.3. Health Risk Assessment

Previous studies indicated that heavy metals can enter the human body through the following four exposure pathways: hand-to-oral ingestion, dermal absorption, inhalation, and food ingestion. In this study, the dose-response model proposed by the USEPA [39,84] was used for the calculation of potential health risk assessment caused by soil heavy metals.

(A3) $$ADI_{ing}=C\times \frac{EF\times ED}{BW\times AT}\times {IR}_{ing}\times CF$$

(A4) $$ADI_{dermal}=C\times \frac{EF\times ED\;}{BW\times AT}\times SA\times AF\times ABS\times CF$$

(A5) $$ADI_{inh}=C\times \frac{EF\times ED\;}{BW\times AT}\times \frac{{IR}_{inh}}{PEF}$$

(A6) $$ADI_{food}=C_{food}\times \frac{EF\times ED\;}{BW\times AT}\times {IR}_{plant}\times CF$$

where $ADI_{ing}$, $ADI_{dermal}$, $ADI_{inh}$, and $ADI_{food}$ correspond to daily average exposure through the four pathways of hand-to-oral ingestion, dermal absorption, inhalation, and food ingestion, respectively. The variables C and $C_{food}$ represent the concentrations of heavy metals in soils and crops (Table A3), and the remaining parameters are comprehensively described in Table A4.

(A7) $$HQ_{ij}=\frac{ADI_{ij}}{RfD_{ij}}$$

(A8) $$HI=\sum {HQ}_{ij}$$

(A9) $${CR}_{ij}={ADI}_{ij}\times {SF}_{ij}$$

(A10) $$TCR=\sum {CR}_{ij}$$

HI and TCR represent the non-carcinogenic and carcinogenic risk indices of various heavy metals across all exposure pathways, respectively. $ADI_{ij}$, $RfD_{ij}$, and ${SF}_{ij}$ denote the ingestion, reference dose, and slope factor of individual heavy metal i through pathway j. Detailed parameters are provided in Table A5. As suggested by previous studies, the values of HI and TCR respectively exceeded 1.0 and $1\times 10^{-4}$, indicating significant potential non-carcinogenic and carcinogenic risks [85,86].

### Appendix A.4. Positive Matrix Factorization

The PMF decomposes the sample data matrix $X_{ij}$ into the residual matrix $E_{ij}$, the score factorial matrix $G_{ij}$, and the factor loading matrix $F_{kj}$ [50]. We used the minimum objective function *Q* to perform calculations and obtain the optimal factor contributions and factor loadings. Unsuitable points were excluded to fit the optimal parameters and define samples with uncertainty ratio residuals greater than 4. The uncertainty data $U_{ij}$ corresponding to the measured data are calculated from the measured values, the detection limit (MDL), and the error coefficient [51].

(A11) $$X_{ij}=E_{ij}+\sum_{k=1}^{n}\left(G_{ki}\times F_{kj}\right)$$

(A12) $$Q=\sum_{i=1}^{n}\sum_{j=1}^{m}{\left(\frac{E_{ij}}{U_{ij}}\right)}^{2}$$

(A13) $$U_{ij}=\left\{\begin{array}{c} \;\;\;\;\;\;\;\;\;\;\;\;\;\;\;\;\frac{5}{6}\times MDL\;\;\;\;\;\;\;\;\;\;\;\;\;\;,0<c<MDL\\ \sqrt{{\left(\delta \times c\right)}^{2}+{MDL}^{2}}\;\;\;,\;c>MDL \end{array}\right.$$

where *i* denotes the sample, *j* denotes the element, and *k* denotes the pollutant source; *δ* is the error coefficient and *c* is the measured concentration of a heavy metal in a single sample. The strength of each heavy metal was determined from the signal-to-noise ratio (S/N) during the calculation process, and the optimal number of factors was determined by the minimum objective function *Q* as well as the residuals and the coefficient of determination [52].

**Table A1:** Heavy metal background values for surface and deep soils.

	Cu	Zn	Ni	Pb	Cr	Cd	As	Hg
Surface	17	47.3	14.4	36	50.5	0.056	8.9	0.078
Deep	20.4	51.7	18.2	40.1	58.9	0.037	10	0.053

Note: Unit: mg/kg; the heavy metal background values were published by the China National Environmental Monitoring Centre in 1990 [53].

**Table A2:** Pollution assessment criteria.

Pollution Index	Contamination Level	Value
Geo-accumulation index (*I_geo_*)	Unpolluted	*I_geo_* ≤ 0
	Slightly to moderately polluted	0 < *I_geo_* ≤ 1
	Moderately polluted	1 < *I_geo_* ≤ 2
	Moderately to heavily polluted	2 < *I_geo_* ≤ 3
	Heavily polluted	3 < *I_geo_* ≤ 4
	Heavily to extremely polluted	4 < *I_geo_* ≤ 5
	Extremely polluted	*I_geo_* > 5
Potential ecological risk index (*Er*, *RI*)	Low pollution	*Er* < 40, *RI* < 150
	Medium pollution	40 ≤ *Er* < 80, 150 ≤ *RI* < 300
	Strong pollution	80 ≤ *Er* < 160, 300 ≤ *RI* < 600
	Very strong pollution	160 ≤ *Er* < 320, 600 ≤ *RI* < 1200
	Extreme pollution	*Er* ≥ 320, *RI* ≥ 1200

**Table A3:** Heavy metal bio-concentration factors for various types of crops.

HMs	Crop Category					
	Leaf Vegetables [87,88]	Fruit Vegetables [87,89]	Root Vegetables [87,89]	Inflorescence Vegetables [90,91,92]	Sugarcane [87,93]	Banana [87,89]
Cu	1.05 × 10^−2^	1.12 × 10^−2^	2.92 × 10^−2^	1.13 × 10^−1^	2.31 × 10^−2^	8.45 × 10^−3^
Zn	1.31 × 10^−2^	2.46 × 10^−2^	3.48 × 10^−2^	7.90 × 10^−2^	2.28 × 10^−2^	4.65 × 10^−3^
Ni	5.70 × 10^−3^	7.50 × 10^−3^	1.38 × 10^−2^	3.60 × 10^−2^	1.14 × 10^−2^	4.55 × 10^−3^
Pb	1.21 × 10^−2^	7.97 × 10^−3^	1.87 × 10^−2^	1.40 × 10^−2^	1.62 × 10^−2^	6.25 × 10^−3^
Cr	9.32 × 10^−3^	3.10 × 10^−3^	5.80 × 10^−3^	1.00 × 10^−2^	5.50 × 10^−3^	1.65 × 10^−3^
Cd	4.95 × 10^−2^	5.57 × 10^−2^	1.89 × 10^−1^	4.23 × 10^−1^	1.07 × 10^−1^	6.35 × 10^−3^
As	2.50 × 10^−3^	1.10 × 10^−3^	3.10 × 10^−3^	2.50 × 10^−4^	5.00 × 10^−4^	7.40 × 10^−3^
Hg	5.37 × 10^−2^	5.20 × 10^−3^	8.00 × 10^−3^	6.10 × 10^−2^	2.52 × 10^−1^	6.40 × 10^−3^

**Table A4:** Exposure dose input parameters for soil heavy metals under different exposure routes.

Parameter	Description	Unit	Type	Children	Adult	Reference
BW	Average body weight	kg	Point	18.2	65 (male) 56.8 (female)	[94,95]
AT	Average time for non-cancer risk	days	Point	365 × ED	366 × ED	[96,97]
	Average time for carcinogenic risk	days	Point	365 × 70		[97]
PEF	Particle emission factor	m^3^/kg	Point	1.36 × 10^9^	1.36 × 10^9^	[97]
ABS	Dermal absorption factor	unitless	Point	0.1 (Cu)	0.02 (Zn)	[39,98]
				0.001 (Ni)		
				0.006 (Pb)	0.001 (Cr)	
				0.14 (Cd)		
				0.03 (As)	0.05 (Hg)	
AF	Skin adherence factor for soil	mg/cm^2^	Lognormal	LN (0.65,1.2)	LN (0.49,0.54)	[97,99]
SA	Exposed skin surface area	cm^2^	Point	2772	5440 (male) 4800 (female)	[94,96]
ED	Exposure duration	years	Point	6	24	[96]
EF	Exposure frequency	days/year	Triangular	Tri(180,345,365)		[96,100]
CF	Conversion coefficient	unitless	Point	1 × 10^−6^		[94,101]
IR_ing_	Ingestion rate of soil	mg/day	Triangular	Tri (66,103,161)	Tri (4,30,52)	[102]
IR_inh_	Inhalation rate	m^3^/day	Point	7.65	20	[97]
IR_food_	Ingestion rate of agricultural products	mg/day	Point	7.5 × 10^4^	1.5 × 105	[101]

**Table A5:** Heavy metal non-carcinogenic reference doses and carcinogenicity slope factors.

Elements	RfD (mg/(kg∙d))			SF ((kg∙d)/mg)			Reference
	Ingestion	Dermal Absorption	Inhalation	Ingestion	Dermal Absorption	Inhalation	
Cu	4.00 × 10^−2^	1.20 × 10^−2^	4.02 × 10^−2^	N/A	N/A	N/A	[100,103,104]
Zn	3.00 × 10^−1^	6.00 × 10^−2^	3.00 × 10^−1^	N/A	N/A	N/A	[100,103,104]
Ni	2.00 × 10^−2^	5.40 × 10^−3^	2.06 × 10^−2^	4.10 × 10^−1^	3.68	8.40 × 10^−1^	[100,103,104,105,106,107]
Pb	3.50 × 10^−3^	5.25 × 10^−4^	3.52 × 10^−3^	8.50 × 10^−3^	8.50 × 10^−3^	4.20 × 10^−2^	[100,103,104,108,109]
Cr	3.00 × 10^−3^	6.00 × 10^−5^	2.86 × 10^−5^	5.00 × 10^−1^	20	42	[103,104,110,111]
Cd	1.00 × 10^−3^	1.00 × 10^−5^	1.00 × 10^−5^	6.10	20	6.30	[84,102,103,104,110]
As	3.00 × 10^−4^	1.23 × 10^−4^	3.01 × 10^−4^	1.50	3.66	15.10	[100,103,104]
Hg	3.00 × 10^−4^	2.10 × 10^−5^	8.57 × 10^−5^	1.5	1.5	15.1	[39,103,104]

Note: RfDing, RfDder, and RfDinh represent the reference doses of HMs by ingestion, dermal absorption, and inhalation, respectively [mg (kg day)^–1^]; SFing, SFder, and SFinh represent the carcinogenicity slope factor of HMs by ingestion, dermal absorption, and inhalation, respectively [(kg day) mg^–1^].

**Table A6:** Calculated hazard indices and carcinogenic risks caused by soil heavy metals based on Monte Carlo simulations.

Risk	HMs	Surface Soil						Deep Soil					
		Mean			10% Cumulative Probability Risk			Mean			10% Cumulative Probability Risk		
		Children	Adult Males	Adult Females	Children	Adult Males	Adult Females	Children	Adult Males	Adult Females	Children	Adult Males	Adult Females
HQ	Cu	2.06 × 10^−1^	1.06 × 10^−1^	1.19 × 10^−1^	4.06 × 10^−3^	−7.55 × 10^−3^	−1.09 × 10^−2^	1.56 × 10^−1^	7.99 × 10^−2^	8.95 × 10^−2^	1.57 × 10^−4^	−7.51 × 10^−3^	−1.06×10^−2^
	Zn	5.27 × 10^−2^	2.78 × 10^−2^	3.16 × 10^−2^	9.18 × 10^−3^	3.44 × 10^−3^	3.70 × 10^−3^	4.32 × 10^−2^	2.27 × 10^−2^	2.58 × 10^−2^	7.13 × 10^−3^	2.48 × 10^−3^	2.68×10^−3^
	Ni	1.79 × 10^−1^	9.45 × 10^−2^	1.08 × 10^−1^	8.25 × 10^−2^	4.07 × 10^−2^	4.66 × 10^−2^	1.48 × 10^−1^	7.79 × 10^−2^	8.91 × 10^−2^	6.61 × 10^−2^	3.23 × 10^−2^	3.69×10^−2^
	Pb	9.31 × 10^−1^	4.77 × 10^−1^	5.42 × 10^−1^	6.22 × 10^−1^	5.02 × 10^−1^	3.04 × 10^−1^	7.90 × 10^−1^	4.04 × 10^−1^	4.60 × 10^−1^	2.44 × 10^−1^	3.45 × 10^−1^	2.76×10^−1^
	Cr	9.83 × 10^−1^	4.57 × 10^−1^	5.15 × 10^−1^	6.30 × 10^−1^	2.61 × 10^−1^	2.91 × 10^−1^	8.18 × 10^−1^	3.79 × 10^−1^	4.28 × 10^−1^	4.91 × 10^−1^	1.99 × 10^−1^	2.22×10^−1^
	Cd	8.37 × 10^−1^	3.80 × 10^−1^	4.01 × 10^−1^	4.26 × 10^−1^	1.66 × 10^−1^	1.62 × 10^−1^	6.55 × 10^−1^	2.96 × 10^−1^	3.12 × 10^−1^	2.96 × 10^−1^	1.30 × 10^−1^	1.34×10^−1^
	As	1.88	7.07 × 10^−1^	7.72 × 10^−1^	7.63 × 10^−1^	9.65 × 10^−2^	7.32 × 10^−2^	1.61	6.11 × 10^−1^	6.69 × 10^−1^	5.51 × 10^−1^	5.31 × 10^−2^	2.96×10^−2^
	Hg	7.15 × 10^−2^	3.45 × 10^−2^	3.77 × 10^−2^	−1.96 × 10^−1^	−1.15 × 10^−1^	−1.34 × 10^−1^	5.15 × 10^−2^	2.50 × 10^−2^	2.75 × 10^−2^	−1.46 × 10^−1^	−8.57 × 10^−2^	−9.94×10^−2^
HI		5.14	2.28	2.53	3.83	1.57	1.71	4.27	1.90	2.10	3.03	1.24	1.36
CR	Ni	1.26 × 10^−4^	2.67 × 10^−4^	3.05 × 10^−4^	5.88 × 10^−5^	1.16 × 10^−4^	1.32 × 10^−4^	1.05 × 10^−4^	2.20 × 10^−4^	2.52 × 10^−4^	4.72 × 10^−5^	9.20 × 10^−5^	1.05×10^−4^
	Pb	2.26 × 10^−6^	4.67 × 10^−6^	5.34 × 10^−6^	1.47 × 10^−6^	2.91 × 10^−6^	3.33 × 10^−6^	1.92 × 10^−6^	3.96 × 10^−6^	4.53 × 10^−6^	1.18 × 10^−6^	2.32 × 10^−6^	2.65×10^−6^
	Cr	1.23 × 10^−4^	2.29 × 10^−4^	2.59 × 10^−4^	7.76 × 10^−5^	1.29 × 10^−4^	1.44 × 10^−4^	1.02 × 10^−4^	1.90 × 10^−4^	2.15 × 10^−4^	6.04 × 10^−5^	9.79 × 10^−5^	1.09×10^−4^
	Cd	1.34 × 10^−4^	2.92 × 10^−4^	3.32 × 10^−4^	−4.50 × 10^−5^	−1.10 × 10^−4^	−1.29 × 10^−4^	1.01 × 10^−4^	2.19 × 10^−4^	2.48 × 10^−4^	−7.05 × 10^−8^	−7.10 × 10^−6^	−9.82×10^−6^
	As	7.24 × 10^−5^	1.09 × 10^−4^	1.19 × 10^−4^	2.95 × 10^−5^	1.50 × 10^−5^	1.13 × 10^−5^	6.20 × 10^−5^	9.44 × 10^−5^	1.03 × 10^−4^	2.13 × 10^−5^	8.22 × 10^−6^	4.61×10^−6^
	Hg	1.67 × 10^−6^	3.52 × 10^−6^	4.01 × 10^−6^	−8.59 × 10^−6^	−1.95 × 10^−5^	−2.23 × 10^−5^	1.24 × 10^−6^	2.63 × 10^−6^	3.00 × 10^−6^	−6.39 × 10^−6^	−1.45 × 10^−5^	−1.66×10^−5^
TCR		4.60×10^−4^	9.05 × 10^−4^	1.02 × 10^−3^	2.57 × 10^−4^	4.51 × 10^−4^	5.04 × 10^−4^	3.73 × 10^−4^	7.30 × 10^−4^	8.26 × 10^−4^	2.43 × 10^−4^	4.42 × 10^−4^	4.97 × 10^−4^

Note: HQ and HI respectively represent hazard quotient of each HM and hazard index posed by multiple HMs; CR and TCR represent carcinogenic risk of each HM and total carcinogenic risk posed by multiple HMs.

**Table A7:** Correlation coefficients between heavy metals for both surface and deep soils.

	Surface	Cu	Zn	Ni	Pb	Cr	Cd	As	Hg
Deep									
Cu			0.547 **	0.753 **	0.044	0.828 **	0.406 **	0.014	0.636 **
Zn		0.871 **		0.616 **	0.482 **	0.539 **	0.429 **	0.390 **	0.530 **
Ni		0.954 **	0.910 **		0.389 **	0.757 **	0.23	0.427 **	0.475 **
Pb		0.510 **	0.827 **	0.657 **		0.23	−0.212	0.732 **	0.065
Cr		0.964 **	0.866 **	0.966 **	0.525 **		0.172	0.301 *	0.578 **
Cd		0.648 **	0.742 **	0.542 **	0.541 **	0.547 **		−0.156	0.429 **
As		0.632 **	0.831 **	0.747 **	0.878 **	0.663 **	0.522 **		0.053
Hg		0.897 **	0.851 **	0.845 **	0.575 **	0.826 **	0.768 **	0.633 **	

Note: ** Indicates a significant correlation at the 0.01 level. * Indicates a significant correlation at the 0.05 level.
